# Dissolved oxygen control strategy for improvement of TL1-1 production in submerged fermentation by *Daldinia eschscholzii*

**DOI:** 10.1186/s40643-016-0134-4

**Published:** 2017-01-02

**Authors:** Xing-chen Wei, Liu Tang, Yan-hua Lu

**Affiliations:** 1 State Key Laboratory of Bioreactor Engineering, East China University of Science and Technology, 130 Meilong Road, Box 283#, Shanghai, 200237 People’s Republic of China; 2 Shanghai Collaborative Innovation Center for Biomanufacturing Technology, 130 Meilong Road, Shanghai, 200237 People’s Republic of China

**Keywords:** TL1-1, Dissolved oxygen control, Submerged fermentation, *Daldinia eschscholzii*

## Abstract

**Background:**

2,3-Dihydro-5-hydroxy-2-methylchromen-4-one (TL1-1) is a phenolic compound with significant anti-fungal and anti-cancer activities produced by *Daldinia eschscholzii* (*D. eschscholzii*). However, studies have rarely been reported on the fermentation process of *D. eschscholzii* due to the urgent demand for its pharmaceutical researches and applications.

**Results:**

In this work, the optimal fermentation medium for improved TL1-1 yield was first obtained in a shake flask. As the fermentation process was scaling up, the marked effects of dissolved oxygen (DO) on cell growth and TL1-1 biosynthesis were observed and confirmed. Controlling a suitable DO level by the adjustment of agitation speed and aeration rate remarkably enhanced TL1-1 production in a lab-scale bioreactor. Moreover, the fermentation of *D. eschscholzii* was successfully applied in 500-L bioreactor, and TL1-1 production has achieved 873.63 mg/L, approximately 15.4-fold than its initial production (53.27 mg/L).

**Conclusions:**

Dissolved oxygen control strategy for enhancing TL1-1 production was first proposed. Furthermore, control of the appropriate DO level has successfully performed for improving TL1-1 yield and scale-up of *D. eschscholzii* fermentation process.

**Electronic supplementary material:**

The online version of this article (doi:10.1186/s40643-016-0134-4) contains supplementary material, which is available to authorized users.

## Background

2,3-Dihydro-5-hydroxy-2-methyl-chrlchromen-4-one (TL1-1) is one kind of phenolic compound isolated from the filamentous fungus *Daldinia eschscholzii* IFB-TL01 (Zhang et al. 2011). According to previous studies, TL1-1 has revealed significant anti-fungal activity against plant pathogenic fungi, *Microbotryum violaceum* (Dai et al. 2006), and it suggests that TL1-1 should be developed as a potential candidate for agricultural antibiotic. It is also reported that it inhibits cell proliferation of various cancer cells (leukemia, melanoma and liver) with IC_50_ values of 8–55 μg/mL. Furthermore, the little cytotoxicity to normal cells indicates that TL1-1 could serve as a promising candidate for cancer treatment (Zilla et al. 2013; Pathania et al. 2015). However, low production would severely restrict its further pharmaceutical researches and applications. Moreover, little research has been reported on the submerged fermentation of *D. eschscholzii*. Therefore, it is urgent to develop an effective strategy to enhance the yield of TL1-1 from fermentation process.

In the fermentation processes, the medium composition and culture conditions play a crucial role in both cell growth and product formation (Guleria et al. 2016). The molasses used as carbon source significantly enhance Fumigaclavine C production (from 75.9 to 215.0 mg/L) by *Aspergillus fumigatus* CY018 in a lab-scale bioreactor (Zhu et al. 2015). Distinguished from shake flask, bioreactor requires for optimal conditions (agitation speed and aeration rate) to achieve the maximum productivity in industrial scale-up level (Zou et al. 2012). To satisfy cell growth and maintain desired product synthesis, it is requested to settle the problems of shear stress, mass transfer and mixing in submerged fermentation process (Lu et al. 2015; Formenti et al. 2014; Werner et al. 2014). In *Cellulophaga lytica* LBH-14 fermentation, cell growth and cellobiase formation are regulated by adjustment of agitation speed as well as aeration rate, and the maximum cellobiase activity (140.1 U/mL) is obtained in 100-L bioreactor, 1.52 times higher than that in shake flask (Gao et al. 2015).

Accumulative studies demonstrate that dissolved oxygen has become a significant factor in the scale-up of industrial aerobic fermentation process (Pollard et al. 2007). Due to inadequate oxygen supply capacity, various technologies are employed to improve DO level (Häusler et al. 2016; Garcia-Ochoa and Gomez 2009). For example, in *Ralstonia eutropha* fermentation, off-gas recycle pressure swing adsorption is applied to provide high-purity oxygen continuously, and higher titer of poly(3-hydroxybutyrate) is acquired in industrial scale (Chang et al. 2010). However, the excessive oxygen concentration caused by increase of agitation speed and aeration rate could have negative impacts on bioprocess because of oxygen toxicity or shear stress for sensitive cells (Nagy 2002). That means the demand for oxygen in microorganisms varies among different fermentation processes. To ensure the appropriate oxygen supplement, DO is commonly controlled at a suitable level (Cao et al. 2013). Take submerged fermentation of *Saccharopolyspora spinosa*, for example, DO is kept at 40–50% during 0–96 h for cell growth and at 25–35% during 96–240 h for spinosad formation (Bai et al. 2015). In consequence, the application of DO control strategy could effectively enhance desired product yield.

In this paper, the optimal medium for higher yield of TL1-1 was first obtained. Subsequently, the significant effects of the oxygen concentration on cell growth and TL1-1 biosynthesis were discovered. In the scale-up process, DO level was further confirmed to have played a critical role in desired product formation. The strategy of controlling the appropriate DO level was conducted through the adjustment of agitation speed and aeration rate, which enhanced TL1-1 production and was scaled up in 500-L bioreactor successfully. The information obtained would contribute to the industrial application of submerged fermentation of filamentous fungus.

## Methods

### Microorganism

The fungus, *D. eschscholzii* IFB-TL01 (CCTCCM 207198) from the *Tenodera aridifolia* gut, was friendly provided by Nanjing University (Zhang et al. 2008). This strain was preserved on potato dextrose agar (PDA) and sub-cultured every month. The strain was incubated on PDA at 28 °C for 7 days. Subsequently, two agar tablets (size of 1.0 cm × 1.0 cm) were cut and inoculated in a 1-L Erlenmeyer flask containing 400 mL seed medium (potato dextrose broth) (PDB) (Pan et al. 2015).

### Optimization of fermentation media in shake flask

Seven fermentation media (Medium 1–Medium 7) (data not shown) were screened for TL1-1 accumulation (Zhang et al. 2008; Atlas 2010; Cai et al. 2009). Subsequently, the carbon sources with same molar amount of C (glucose, sucrose, mannitol and soluble starch) were chose to investigate the influence of biomass and TL1-1 production in M5.

The details of culture conditions including inoculation ages, inoculums volume, and initial pH of fermentation medium were optimized in our previous work (data not shown and detailed data in Additional file 1: Figure S1). For the shake flask fermentation, the seed was cultured at 180 rpm, 28 °C for 72 h. Subsequently, 7 mL seed broth was inoculated into a 250-mL shake flask with 50 mL fermentation medium of which initial pH was 9.0 ± 0.2 (before sterilization). The above fermentations for TL1-1 production were performed at 180 rpm, 28 °C for 144 h in a shake flask.

### Investigation of fermentation conditions in shake flask

In the test of shaking speed, fermentation was performed for 144 h at 28 °C and 80, 180 and 280 rpm, respectively. To investigate effects of shear stress, 0, 2, 6 and 10 glass beads (diameter of 2 mm) were added into fermentation medium before sterilization. In the study on various oxygen concentrations, the seed broth prepared was transferred into a 250-mL shake flask with 30, 50, 70, 90 and 150 mL medium, respectively. Additionally, 200% silicone oil, 1% *n*-dodecane and 3% *n*-dodecane were added into 50 mL fermentation medium before sterilization, respectively. All data obtained in this work were the mean of triplicate experiments, and the error bars indicated the corresponding standard deviation.

### Optimization of agitation speed in 5-L bioreactor

Optimization of agitation speed was performed in 5-L stirred bioreactor equipped with two layers of six flat-blade disk turbine impeller based on the optimal fermentation medium. 400 mL seed broth, prepared under the same conditions as previously described, was inoculated into the 5-L bioreactor containing 2.9 L medium. The fungus was cultivated at 28 °C for 120 h with aeration rate of 1.2 air volume/culture volume/min (VVM) during the fermentation. The agitation speed was set as 180, 230 and 280 rpm, respectively. Antifoam (mixture of organic polyether dispersions) was added with 0.08% (v/v). Samples were taken at an interval of 12 h.

### Adjustment of aeration rate in 50-L bioreactor

Adjustment of aeration rate was operated in a 50-L stirred bioreactor equipped with three turbine impellers (Zhang et al. 2014). Based on the amplified standard of similar blade and linear velocity, agitation speed was set at 130 rpm in 50-L bioreactor. The aeration rate was set as 0.6, 0.9 and 1.2 VVM, respectively. Samples were taken at an interval of 24 h.

### Scale-up of the fermentation process

4 L preliminary seed broth (prepared under the same conditions as previously described) was transferred into a 50-L bioreactor containing 40 L PDB; secondary seed was cultivated at 28 °C for 24 h (agitation speed of 150 rpm and aeration rate of 1.0 VVM). Then, the secondary seed broth was inoculated into a 500-L stirred bioreactor equipped with three turbine impellers at inoculums ratio of 14% (v/v). The fungus was cultivated at 28 °C for 192 h with agitation speed of 70 rpm and aeration rate of 0.9 VVM during the fermentation process. Samples were taken at an interval of 24 h.

### Measurement of fungus growth, residual sugar and TL1-1 production

Fungus growth of *D. eschscholzii* was determined by dry cell weight (DCW) (Yang et al. 2016), and residual sugar (mannitol) was determined by the colorimetric assay (Bok and Demain 1977).

The whole fermentation broth was pretreated as report (Zhu et al. 2014). The qualitative and quantitative measurements of TL1-1 were analyzed by HPLC (Agilent 1290 Infinity, DAD-G4212B, USA) with a ZORBAX Eclipse SB-C18 column (4.6 × 250 mm, 5 µm). Each injected sample (20 µL) was eluted with a mobile phase made up of methanol/water (60:40) for 25 min. The flow rate was set as 1.0 mL/min, the operating temperature 25 °C, and the detection wavelength 272 nm, respectively. The standard calibration curve equation was *Y* = 42.95*X* − 341.77 with high linearity in the range of 62.5–750.0 mg/L (linear relative coefficients up to 0.9999), where *Y* is the peak area and *X* is the concentration of standard TL1-1 (mg/L) (data not shown and detailed data in Additional file 1: Figure S2).

## Results and discussion

### Effects of fermentation medium on production of TL1-1

**Fig. 1:**
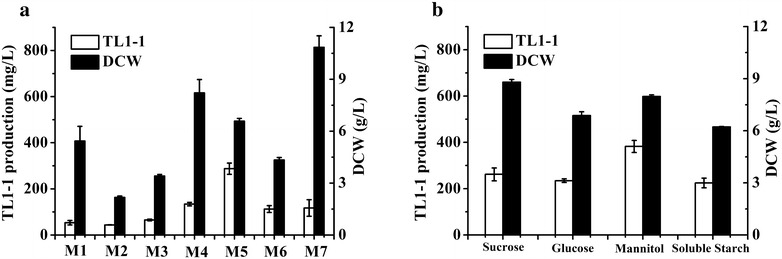
TL1-1 production and DCW (180 rpm, 28 °C) at 144 h in shake flask. **a** In different media (M1–M7) and **b** in different carbon sources with same molar amount of C in M5. *Error bars* show standard deviation of three replicates

Due to low concentration of TL1-1 in M1 which was used for isolation of natural product by *D. eschscholzii*, it is expected to obtain a fermentation medium suitable for higher yield of TL1-1 through selection of seven media and optimization of carbon source. As shown in Fig. 1a and b, M5 selected as basic medium and mannitol (32 g/L) used as suitable carbon source increased TL1-1 production to 382.09 mg/L, compared to that in M1 (53.27 mg/L).

### Effects of fermentation conditions on cell growth and TL1-1 production in shake flask

**Fig. 2:**
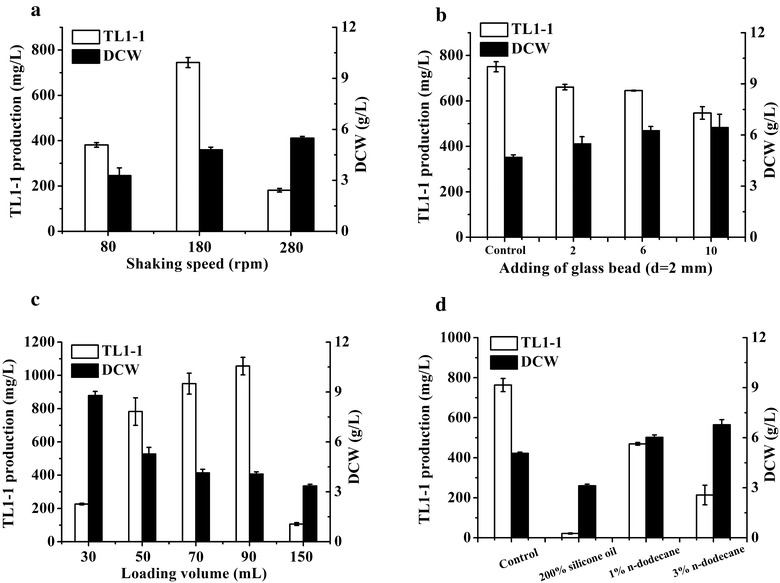
TL1-1 production and DCW at 144 h in shake flask. **a** In different shaking speeds. **b** In different adding numbers of glass beads. **c** In different loading volumes. **d** In different adding concentrations of *n*-dodecane and silicone oil. *Error bars* show standard deviation of three replicates

Oxygen concentration and shear stress were key factors in metabolites synthesis (Wang et al. 2010; Cai et al. 2014). To investigate the effects of oxygen concentration and shear stress on TL1-1 biosynthesis, shaking speed experiment was preformed preliminarily. The data in Fig. 2a clearly showed that biomass (5.48 g/L) was the largest under shaking speed at 280 rpm and the highest TL1-1 production (745.01 mg/L) was obtained at 180 rpm. Compared to 180 rpm, TL1-1 yield was reduced by 48.8 and 75.7% under shaking speed at 80 and 280 rpm, respectively. It was observed that the obvious differences in TL1-1 production existed among different shaking speeds. The explanation may be that the lowest speed could cause insufficient oxygen concentration while highest one may create shear stress, both of which may have affected target product synthesis (Xu and Zhong 2011; Xia et al. 2014).

According to previous reports, high shear stress environment may be stimulated by the addition of glass beads in shake flask (Dobson et al. 2008). To study the effects of shear force on TL1-1 synthesis, the test of glass beads addition was performed. The data showed that total titer of TL1-1 (750.60 mg/L) was the highest without addition of glass beads, and it declined with the addition amounts of glass beads being increased. TL1-1 concentration was decreased by only 27.2% when 10 glass beads were added (Fig. 2b). This indicated that shear stress had a negative effect on TL1-1 biosynthesis to a certain extent and another critical factor may be to explore.

Previous studies showed oxygen concentration was declined with the increase of medium volume in shake flask due to change of oxygen transfer area (Dou et al. 2013). To study the effects of oxygen concentration on TL1-1 biosynthesis, the test of loading volume was operated. It was observed that the increase of fermentation medium volume led to biomass reduction (Fig. 2c). And TL1-1 production was greatly improved in the range of filling volume from 30 to 90 mL, while sharply decreased from 90 to 150 mL (Fig. 2c). To further confirm the effects of oxygen concentration on TL1-1 synthesis, *n*-dodecane and silicone oil were added into fermentation medium before sterilization. Previous studies illustrated that *n*-dodecane as oxygen vector could enhance volumetric mass transfer coefficients to improve oxygen concentration, and a significant amount of silicone oil could isolate oxygen to reduce oxygen concentration during fermentation process (Da Silva et al. 2006; Xu et al. 2015). As shown in Fig. 2d, compared to control (763.49 mg/L and 5.06 g/L), TL1-1 production was significantly declined to 469.35 mg/L and biomass was increased to 6.77 g/L when adding 3% *n*-dodecane. Additionally, double volume of silicone oil was added into medium, both TL1-1 production and biomass were reduced by 97.2 and 38.4%, respectively. These results evidently indicated that higher oxygen concentration was beneficial for cell growth but failed to get higher TL1-1 yield, while extremely low one inhibited both. Oxygen concentration stress played a critical role on TL1-1 biosynthesis. Increasing studies showed many secondary metabolite syntheses were sensitive to oxygen concentration, such as cephamycin C, tylosin and erythromycin (Pollard et al. 2002). Hence, different shaking speeds mainly caused the change of oxygen concentration, as well as shear stress as a secondary factor, which resulted in variation of TL1-1 production.

### Effects of DO on cell growth and TL1-1 production by optimization of agitation speed in 5-L bioreactor

**Fig. 3:**
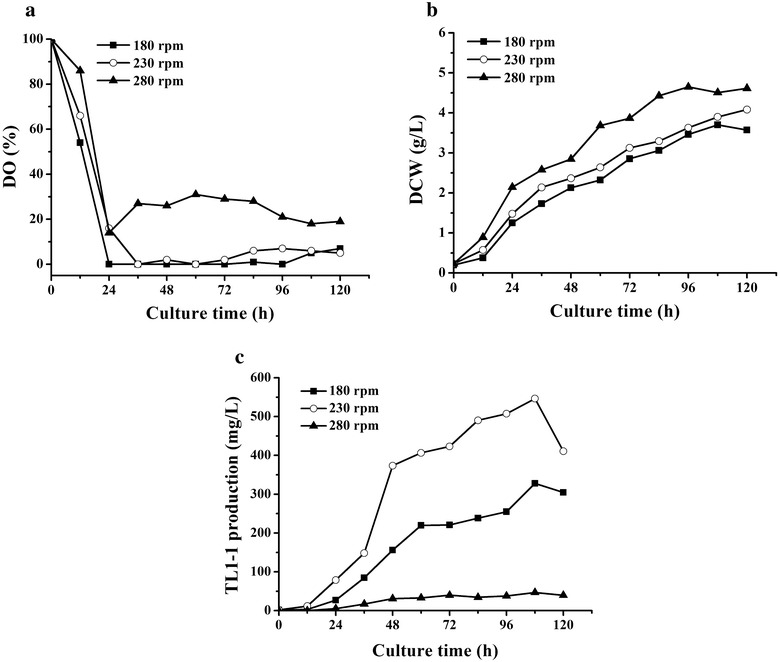
Comparison between the kinetic profiles of **a** DO, **b** DCW and **c** TL1-1 production under aeration rate of 1.2 VVM and agitation speed of 180, 230 and 280 rpm in 5-L bioreactor, respectively

Dissolved oxygen was an essential parameter in bioreactor and the level of DO got improved with the increase of agitation speed and aeration rate in fermentation process (Wang et al. 2010). In 5-L bioreactor, agitation speed was optimized at the same aeration rate (1.2 VVM). The data demonstrated that DO was increased with the enhancement of stirring speed in Fig. 3a. Figure 3b indicates biomass was increased with the improvement of agitation speed, and higher oxygen concentration was beneficial to cell growth significantly. Figure 3a shows that DO at 280 rpm (higher than 20%) was higher than that at 180 and 230 rpm during 24–120 h. And TL1-1 production (46.74 mg/L) was the lowest, correspondently in Fig. 3c. It can be also observed that DO was maintained at a lower level during 24–120 h in Fig. 3a and higher desired production (327.94 and 546.39 mg/L, respectively) was obtained in Fig. 3c at 180 and 230 rpm. However, the minimal agitation speed (180 rpm) resulted in lower concentration of TL1-1 (327.94 mg/L) (Fig. 3c). It is possible that growth of aerobic microorganism may be inhibited under low-oxygen stress environment. The above results were in accordance with shake flake tests (Fig. 2a, c, d). Similar results also occurred in fermentation of *Kluyvera cryocrescens*. Oxygen concentration at high level was disadvantageous to ethanol accumulation. And sufficient oxygen condition accelerated the generation of ATP by reducing NADH, promoting biomass formation. Consequently, more carbon flux was driven to cell growth while less one for ethanol synthesis (Choi et al. 2011). TL1-1 production reached 546.39 mg/L with agitation speed of 230 rpm and aeration rate of 1.2 VVM in 5-L bioreactor. These findings further revealed that DO level was a key factor in TL1-1 formation.

### Effects of DO on cell growth and TL1-1 production by adjustment of aeration rate in 50-L bioreactor

**Fig. 4:**
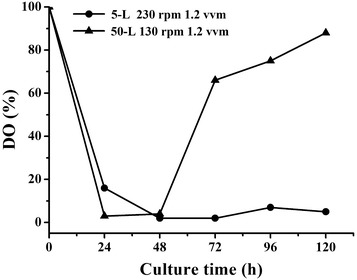
Comparison between the kinetic profiles of DO in 5-L bioreactor (agitation speed 230 rpm and aeration rate 1.2 VVM) and 50-L bioreactor (agitation speed 130 rpm and aeration rate 1.2 VVM)

**Fig. 5:**
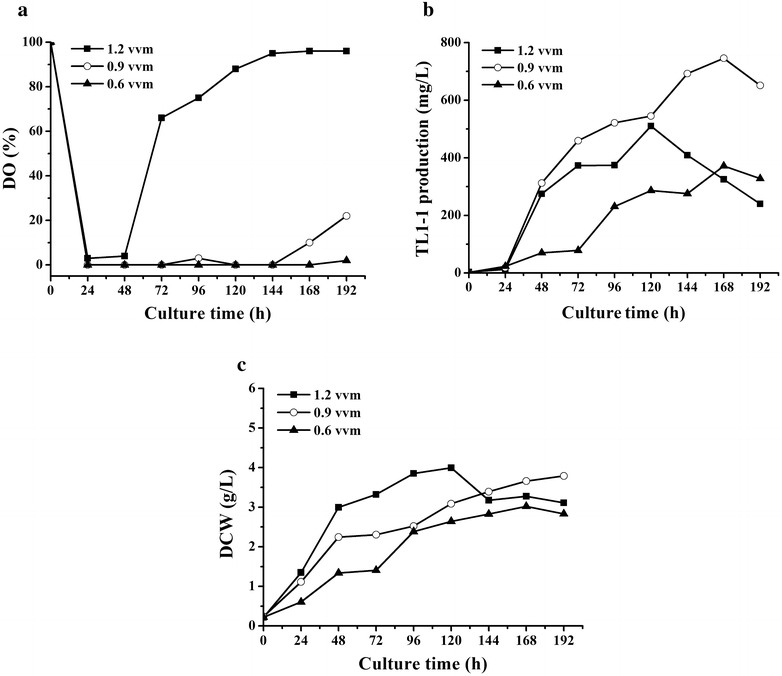
Comparison between the kinetic profiles of **a** DO, **b** TL1-1 production and **c** DCW under agitation speed of 130 rpm and aeration rate of 1.2, 0.9 and 0.6 VVM in 50-L bioreactor, respectively

The fermentation process of *D. eschscholzii* was scaled up to 50-L bioreactor with agitation speed set at 130 rpm and aeration rate at 1.2 VVM. However, the obtained TL1-1 yield (510.13 mg/L) in 50-L bioreactor was lower than that in shake flake. The interesting findings that DO rapidly reached a high level (approximately 60–100%) after 48 h in 50-L bioreactor while maintained a low level in 5-L bioreactor (agitation speed 230 rpm and aeration rate 1.2 VVM) were observed in Fig. 4. And the reason was that oxygen supply capacity in 50-L bioreactor was considerably greater than 1 in 5-L bioreactor. Under higher oxygen concentration, biomass and production reduced after 120 h in 50-L bioreactor (Fig. 5b, c). The explanation may be that the faster growth rate and the stronger intensity of cell metabolism in exponential phase led to the declined strain vitality, shortening target product synthesis phase. Obviously, higher DO level was adverse for TL1-1 accumulation.

Based on our previous results, to control the relatively low DO level, the strategy of reducing the aeration rate was applied to 50-L bioreactor for further improvement of TL1-1 production. The aeration rate was adjusted from 1.2 to 0.6 VVM at the same agitation speed (130 rpm) in 50-L bioreactor. When aeration rate was declined from 1.2 to 0.9 VVM, the value of DO fell to less than 10% during 48–168 h in Fig. 5a and TL1-1 concentration reached 745.58 mg/L at 168 h in Fig. 5b, correspondingly. TL1-1 production was equivalent to shake flake level. Similarly, in *Streptomyces griseorubens* fermentation, DO was kept at a constant level by adjusting agitation speed and aeration rate, which accelerated cellulase and xylanase formation (Zhang et al. 2016). In the next study, aeration rate was decreased from 0.9 to 0.6 VVM, and DO was maintained constant afterward at an extremely low level during 24–192 h (Fig. 5a). It was observed that cell growth got restricted under oxygen-limited conditions in Fig. 5c and TL1-1 yield was decreased to 371.72 mg/L in Fig. 5b. The cell physiologic state has been recognized to have impacts on secondary metabolisms of microorganisms. For example, the cell growth of *Porphyridium purpureum* was significantly inhibited and the production of total fatty acids as well as arachidonic acid was declined with limited amount of oxygen (Su et al. 2016). The level of oxygen available played an important role in control of the competition between the biomass formation and product synthesis. These operating conditions (agitation speed 130 rpm and aeration rate 0.9 VVM) in 50-L bioreactor seemed to be favorable for TL1-1 formation and the hypothesis that DO control strategy promoted target product biosynthesis was further confirmed.

### Scale-up TL1-1 production in 500-L bioreactor

**Fig. 6:**
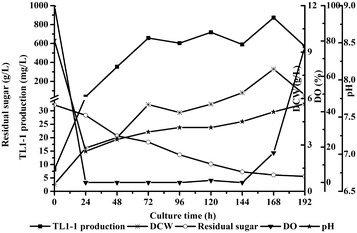
Time courses of residual sugar, TL1-1 production, DCW, DO and pH under agitation speed of 70 rpm and aeration rate of 0.9 VVM in 500-L bioreactor

*Daldinia eschscholzii* fermentation process was performed in 500-L bioreactor based on the optimized medium. With sugar consumed, cell grew and TL1-1 synthesized continuously during 0–168 h in Fig. 6. In 500-L bioreactor, DO was also kept among 0 to 10% during 24–168 h under the operating conditions (agitation speed 70 rpm and aeration rate 0.9 VVM). TL1-1 production achieved 873.63 mg/L at 168 h, which surpassed the shake flake level (763.24 mg/L). Accordingly, the fermentation process of *D. eschscholzii* in 500-L bioreactor was established successfully. It was proved that the strategy of appropriate control of DO significantly improved TL1-1 yield.

## Conclusions

In this study, the fermentation medium and conditions for high yield of TL1-1 were first attained. In the scale-up process, the correlation between oxygen concentration and TL1-1 synthesis was discovered and discussed. The present study suggested higher production should be obtained under the appropriate DO level. The DO control strategy by adjusting agitation speed and aeration rate was proposed and significantly enhanced TL1-1 yield in bioreactor. Furthermore, the fermentation process of *D. eschscholzii* was successfully scaled up to 500-L bioreactor and TL1-1 production has achieved 873.63 mg/L, approximately 15.4-fold than its initial one (53.27 mg/L). This work demonstrated that DO control strategy was an economic and effective approach for enhancement of TL1-1 productivity and would be helpful in the common large-scale submerged fermentation process of filamentous fungus.

## Additional file

**Additional file 1: Figure S1.** TL1-1 production and DCW at 144 h in shake flask. (a) In different inoculation ages. (b) Inoculums volumes. (c) In different initial pH of fermentation medium. **Figure S2.** The HPLC chromatogram of (a) TL1-1 standard and (b) fermentation broth extract at the detection wavelength of 272 nm.

## Abbreviations

TL1-1: 2,3-dihydro-5-hydroxy-2-methylchromen-4-one
PDA: potato dextrose agar
PDB: potato dextrose broth
VVM: air volume/culture volume/min
*D. eschscholzii*: *Daldinia eschscholzii* IFB-TL01 (CCTCCM 207198)
M1–M7: Medium 1–Medium 7
DO: dissolved oxygen
DCW: dry cell weight
