# Supplementary material for: Dissolved oxygen control strategy for improvement of TL1-1 production in submerged fermentation by Daldinia eschscholzii
Source: Bioresour Bioprocess. 2017 Jan 2;4(1):1. doi: 10.1186/s40643-016-0134-4 (PMC5236084; doi:10.1186/s40643-016-0134-4)
Supplement: Supplementary file 1 — Additional file 1: Figure S1. TL1-1 production and DCW at 144 h in shake flask. (a) In different inoculation ages. (b) Inoculums volumes. (c) In different initial pH of fermentation medium. Figure S2. The HPLC chromatogram of (a) TL1-1 standard and (b) fermentation broth extract at the detection wavelength of 272 nm. [file 40643_2016_134_MOESM1_ESM.docx]

**Supporting Data**

**Dissolved oxygen control strategy for improvement of TL1-1 production in submerged fermentation by *Daldinia eschscholzii***

Xing-chen Wei^1,2^, Liu Tang^1,2^ and Yan-hua Lu^1,2*^

The components of seven fermentation media (M1-M7) were as follows.

M1: 20.0 g sucrose, 20.0 g malt extract and 1.0 g peptone.

M2: 2.0 g glucose, 2.0 g cornmeal and 2.0 g yeast extract powder.

M3: 6.0 g glucose, 6.0 g malt extract, 1.8 g maltose and 1.2 g yeast extract powder.

M4: contained 30.0 g glucose, 3.0 g malt extract, 3.0 g yeast extract powder, 5.0 g peptone and 5.0 g CaCO_3_.

M5: 30.0 g sucrose, 5.0 g yeast extract powder, 3.0 g NaNO_3_, 1.0 g K_2_HPO_4_·3H_2_O, 0.5 g KCl, 0.5 g MgSO_4_·7H_2_O and 0.01 g FeSO_4_·7H_2_O.

M6: 10.0 g glucose, 1.0 g yeast extract powder, 0.5 g K_2_HPO_4_·3H_2_O, 0.25g MgSO_4_·7H_2_O and 0.5 mL of FeCl_3_ (0.1%).

M7: 40.0 g soluble starch, 40.0 g sucrose, 30.0 g maltose, 2.0 g peptone, 1.0 g yeast extract, 0.5 g K_2_HPO_4_, 0.5 g soybean meal and 0.3 g MgSO_4_·7H_2_O.

Each medium was dissolved by 1.0 L distilled water.

**Figure**

**a**


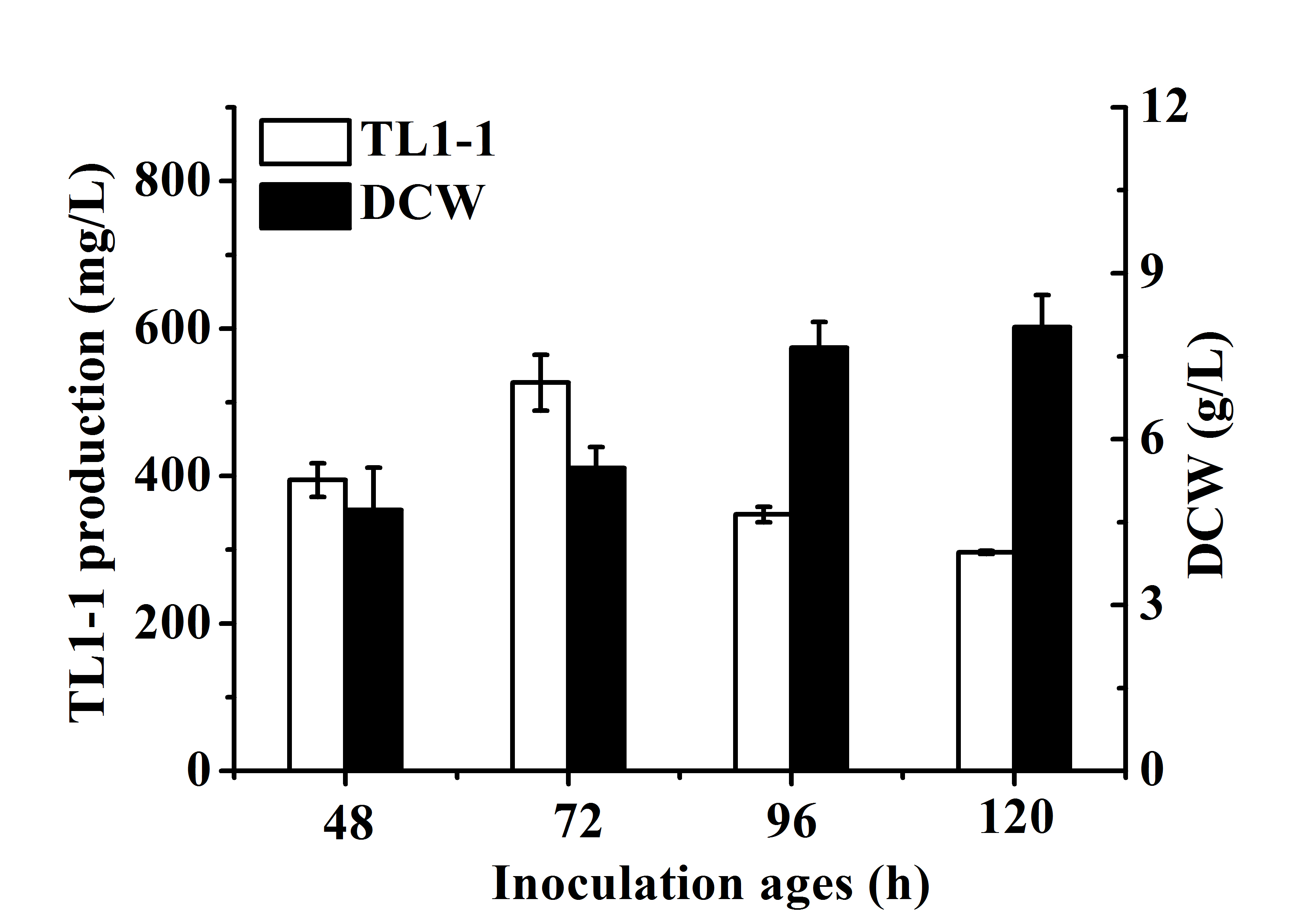


**b**


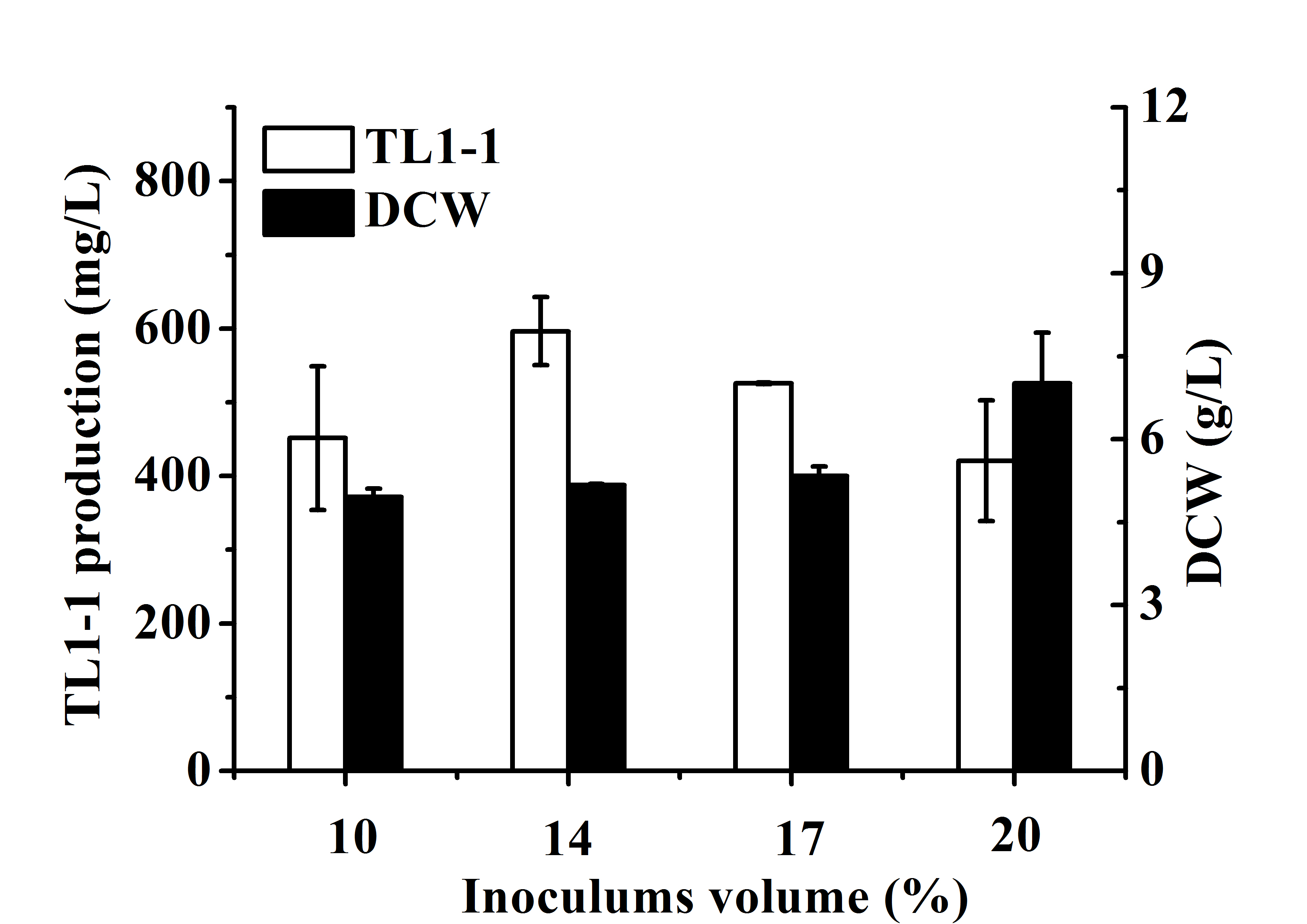


**c**


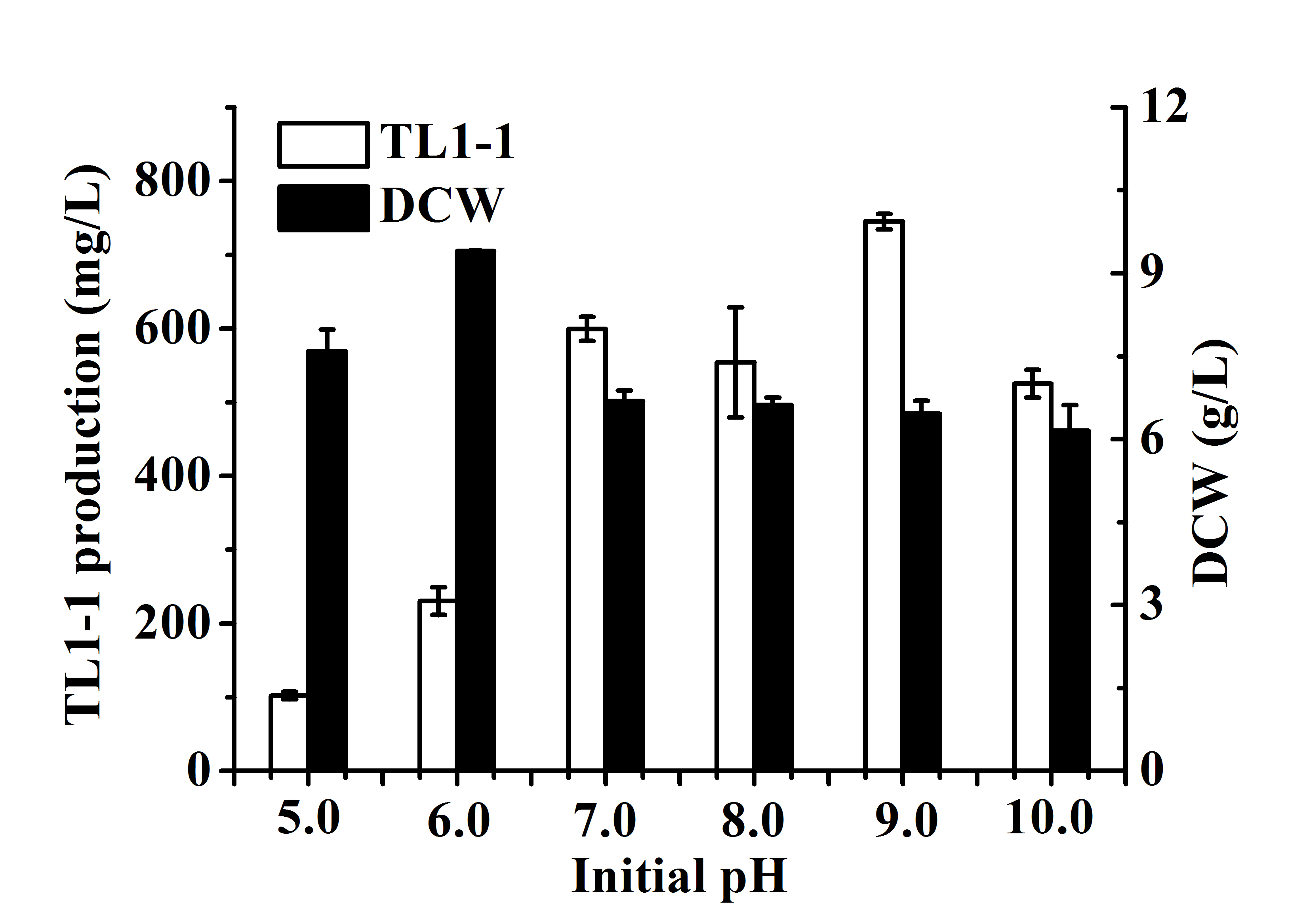


**Fig. S1** TL1-1 production and DCW at 144 h in shake flask. (a) in different inoculation ages. (b) inoculums volumes. (c) in different initial pH of fermentation medium.

When the inoculation age was 72 h, inoculums volume was 14% (v/v), and initial pH value was 9.0 ± 0.2 (before sterilization), TL1-1 production reached to 763.24 mg/L (Fig. S1).

TL1-1 standard

a

TL1-1

fermentation broth extract

b

**Fig. S2** The HPLC chromatogram of (a) TL1-1 standard and (b) fermentation broth extract at the detection wavelength of 272 nm.
